# FOXO 1 deletion in chondrocytes rescues diabetes-impaired fracture healing by restoring angiogenesis and reducing apoptosis

**DOI:** 10.3389/fendo.2023.1136117

**Published:** 2023-08-02

**Authors:** Mohammed A. Alharbi, Dana T. Graves

**Affiliations:** ^1^ Department of Endodontics, Faculty of Dentistry, King Abdulaziz University, Jeddah, Saudi Arabia; ^2^ Department of Periodontics, School of Dental Medicine, University of Pennsylvania, Philadelphia, PA, United States

**Keywords:** animal model, bone, inflammation, neovascularization, FOXO, T1DM, cartilage, apoptosis

## Abstract

**Introduction:**

Diabetes mellitus is associated with higher risks of long bone and jaw fractures. It is also associated with a higher incidence of delayed union or non-union. Our previous investigations concluded that a dominant mechanism was the premature loss of cartilage during endochondral bone formation associated with increased osteoclastic activities. We tested the hypothesis that FOXO1 plays a key role in diabetes-impaired angiogenesis and chondrocyte apoptosis.

**Methods:**

Closed fractures of the femur were induced in mice with lineage-specific FOXO1 deletion in chondrocytes. The control group consisted of mice with the FOXO1 gene present. Mice in the diabetic group were rendered diabetic by multiple streptozotocin injections, while mice in the normoglycemic group received vehicle. Specimens were collected 16 days post fracture. The samples were fixed, decalcified, and embedded in paraffin blocks for immunostaining utilizing anti cleaved caspase-3 or CD31 specific antibodies compared with matched control IgG antibody, and apoptosis by the TUNEL assay. Additionally, ATDC5 chondrocytes were examined in vitro by RT-PCR, luciferase reporter and chromatin immunoprecipitation assays.

**Results:**

Diabetic mice had ~ 50% fewer blood vessels compared to normoglycemic mice FOXO1 deletion in diabetic mice partially rescued the low number of blood vessels (p < 0.05). Additionally, diabetes increased caspase-3 positive and apoptotic chondrocytes by 50%. FOXO1 deletion in diabetic animals blocked the increase in both to levels comparable to normoglycemic animals (p < 0.05). High glucose (HG) and high advanced glycation end products (AGE) levels stimulated FOXO1 association with the caspase-3 promoter in vitro, and overexpression of FOXO1 increased caspase-3 promoter activity in luciferase reporter assays. Furthermore, we review previous mechanistic studies demonstrating that tumor necrosis factor (TNF) inhibition reverses impaired angiogenesis and reverses high levels of chondrocyte apoptosis that occur in fracture healing.

**Discussion:**

New results presented here, in combination with recent studies, provide a comprehensive overview of how diabetes, through high glucose levels, AGEs, and increased inflammation, impair the healing process by interfering with angiogenesis and stimulating chondrocyte apoptosis. FOXO1 in diabetic fractures plays a negative role by reducing new blood vessel formation and increasing chondrocyte cell death which is distinct from its role in normal fracture healing.

## 1 Introduction

Fracture healing is a complex process that requires well-orchestrated and coordinated events that involve various cell types (1). An early and critical step in healing is the proliferation and recruitment of mesenchymal stem cells and their differentiation to chondrocytes, osteoblasts, and other cell types. Chondrocytes lay down cartilage to support and stabilize the fracture site (2). Our lab has recently shown that chondrocytes modulate osteoclastogenesis by producing receptor activator of nuclear factor kappa-β ligand (RANKL), which stimulates the removal of cartilage during the early stages of endochondral bone formation (3). This process coincides with neovascularization, which is essential for healing to progress and is negatively modulated by diabetes (4, 5). Consistent with this observation is significantly improved fracture healing with vascular endothelial growth factor (VEGF) stimulated angiogenesis (6). The formation of new blood vessels during fracture healing provides essential nutrition to the callus, helps recruit osteoclast precursors to resorb the cartilage and endothelial cells produce mediators to modulate the healing process (7). Cartilage resorption and chondrocyte apoptosis are critical steps in the transition from a cartilaginous callus to a hard bony callus needed for fracture healing (2, 3, 8).

Diabetes significantly interferes with the fracture repair process (9). Type 1 diabetes mellitus (T1DM) and T2DM are serious concerns worldwide as the incidence of both chronic diseases has increased in the past 20 years. Today it is estimated that 415 million people worldwide are diagnosed with diabetes (10). Diabetes is characterized by high blood glucose levels (hyperglycemia) due to insulin deficiency or due to the inability to respond to insulin. Several animal and human studies demonstrate that T1DM has an impact on bone with a 2-fold increase in fractures compared to non-diabetics (11), and T2DM patients have a 5-fold greater risk of vertebral fracture (12). Moreover, T1DM and T2DM impair the fracture healing process in humans and in diabetic animals (11, 13–15) and increases the risk of bone fracture (16–18). Furthermore, it delays, impairs, and increases the incidents of non-unions in both animals and humans (13, 19). Diabetes delays fracture healing in the jaw bones and is associated with increased mandibular fracture healing complications and increased recurrence of fractures (20, 21).

FOXO1 is a forkhead transcription factor box O family member, which regulates various cellular events, including proliferation, differentiation, apoptosis, and the response to oxidative stress (22). FOXO1, compared to the three other FOXO family members is more highly expressed in cartilage and bone and has a more dramatic impact on these tissues when deleted (23). FOXO1 has several important functions in chondrocytes. It has been recently shown that FOXO1 regulates chondrocyte homeostasis in a FOXO1 loss of function mouse (Agc1- CreERT2;FoxO1f/f) model. These mice exhibit histologic changes collectively indicative of an increased catabolic state (24). FOXO1 also maintains articular chondrocyte homeostasis through the induction of anabolic and autophagy-related gene expression. FOXO1 has a paramount role in protecting chondrocytes against oxidative stress via the ALK5–SMAD3 pathway (25). Additionally, FOXO1 regulates the expression of interleukin 6 (IL-6) in chondrocytes, which is a potent inflammatory mediator regulating (26). Animals with FOXO1 knockout in chondrocytes initially had more cartilage produced and later had greater loss of cartilage. The latter was associated with increased IL-6. These findings suggest that FOXO1 limits the early expansion of cartilage and prevents its loss at a later stage. Additionally, FOXO1 regulates proteoglycan4(Prg4) expression which is crucial fo rmaintaining cartilage integrity (27). Prg4KO mice showed a significan tincrease in chondrocytes apoptosis and cell loss (28, 29).

In the oral cavity, FOXO1 has been reported to play a pivotal role in temporomandibular joint (TMJ) osteoarthritis. When FOXO1 is inhibited by protein kinase B also known as Ak strain transformin (Akt), there is greater extracellular matrix (ECM) degradation as well as increased chondrocyte apoptosis in a TMJ osteoarthritis model (30). This findings suggests that Foxo1 plays a protective role in TMJ osteoarthritis.

We have shown that FOXO1 plays a positive role in promoting chondrocyte function to facilitate normal fracture healing. When FOXO1 is deleted in chondrocytes (Col2a1.Cre**^+^**.FOXO1^L/L^) in normal conditions, there is a reduction in blood vessel formation and a reduction in the capacity of chondrocytes to induce microvascular endothelial cell tube formation *in vitro* (31). This can be mechanistically explained by the significant reduction in the VEGFA expression by chondrocytes upon FOXO1 deletion in these cells. The results are further supported by evidence that FOXO1 binds to the VEGFA promoter in chondrocytes and FOXO1 induces VEGFA transcriptional activity (31).

*In vivo* results point to the importance of FOXO1 activity in chondrocytes in stimulating normal fracture healing and ultimately endochondral bone formation in adult animals. However, in diabetic fracture healing, FOXO1 plays a negative role in the healing process (3, 32). The goal of this report is to identify mechanisms by which FOXO1 can have a negative role in fracture repair through its detrimental impact on chondrocyte function.

## 2 Materials and methods

### 2.1 Animals and diabetes induction

All animal studies were carried out with approval from the University of Pennsylvania.

Institutional Animal Care and Use Committee (IACUC) (Protocol # 803894) and the Guide for the care and use of laboratory animals, eighth edition (2011), were followed. FOXOL/L mice were provided by R.A. DePinho (MD Anderson Cancer Center, Houston, TX) and created as previously described (33). The experimental group included mice with lineage-specific FOXO1 deletion in chondrocytes (Col2α1Cre^+^.FOXO1^L/L^) and results were compared with Col2α1Cre^-^.FOXO1^L/L^ littermate controls. All animals were monitored daily by University Laboratory Animal Services (ULAR) and cages were changed weekly with 5001 Rodent Diet. (Purina Lab Diet, St. Louis, MO). Every cage contained two to five mice under standard conditions with 14-hours light/10-hours dark cycles. Prior to starting the experiment, genotyping was performed via PCR using both Cre and FOXO1 primers using genomic DNA extracted from the mice’s tails/ears. The results were also verified at the end of the experiment. Type-1 diabetes was induced as described by us (34) and developed originally by Like and Rossini (35). Intraperitoneal (IP) streptozotocin (STZ) injections (40 mg/kg; Sigma-Aldrich, St. Louis, Missouri, US) in 10mM citrate buffer were given once every day for five consecutive days. Vehicle alone was used for control mice. Ten days after the last injection, blood glucose levels were measured weekly from small lacerations in the mice tails from both groups. Mice with two consecutive blood glucose readings of >220 mg/dl were considered diabetic. The mice had been diabetic for at least three weeks prior to starting the experiment.

### 2.2 Fractures induction and sample preparations

At 12-14 weeks old, a simple transverse fracture was induced. as previously described (36). Briefly, an incision was made in the knee and a 30-gauge spinal needle was inserted for fixation. A controlled, closed, simple, transverse fracture was created by blunt trauma to the middle of the femur at the mid-diaphyseal region. There was no change in the animal behavior noticed between the different groups. Animals were euthanized, and femurs were harvested 16 days after fracture. All the sites were evaluated radiographically and physically at euthanasia. Fractures that were not mid-diaphyseal or that were grossly comminuted were excluded from the experiment. Less than 5% of the samples were excluded. Fractured samples were fixed for 24 hours in cold 4% paraformaldehyde. Decalcification was achieved by incubating the samples at room temperature in 10% ethylenediaminetetraacetic acid (EDTA) solution for five weeks before embedding them in paraffin blocks. Transverse sections were prepared as described by us and initially by Gerstenfeld et al. (14, 36).

### 2.3 Immunofluoresence

After dewaxing and hydration, a pressure cooker (2100-Retriever Aptum, Southampton, UK) was used at 120°C for 20 minutes with 10mM of citric acid with a pH of 6.0 for antigen retrieval followed by non-specific binding blocking for 55 minutes, using nonimmune serum matching the secondary antibody. Slides were incubated overnight with CD-31 specific antibody (Abcam, ab28364), anti-cleaved caspase 3 antibody (Cell Signaling Technology 96615) or the appropriate isotype-matched negative control IgG (Vector, I-1000, Burlingame, CA). The primary antibody was localized by a biotinylated secondary antibody (Vector, BA-1000). To localize the antibody complex, Alexa Fluor 546–conjugated streptavidin (Invitrogen S-11225, Carlsbad, CA) was used. Nuclei were counterstained with DAPI (Sigma-Aldrich, St. Louis, MO). Images were captured at different magnifications (40x, 200x, and 400x magnification) using a fluorescence microscope (ECLIPSE 90i; Nikon). The exposure time was set so that the IgG control had no signal. The quantification was performed with the aid of NIS Elements AR image analysis software. The unit of measure was the animal. Each value was calculated by examining six to eight animals in each group.

### 2.4 TUNEL assay

Apoptotic cells were detected by DeadEnd™ Colorimetric TUNEL System (Promega, WI, USA), which detects apoptotic cells by labeling and detecting DNA strand breaks by the TUNEL method. To distinguish apoptotic chondrocytes from other cells, the images were combined with a bright-field channel to confirm the cell morphology. In addition, the TUNEL-positive cells were compared with a safranin-o/fast green stain to verify the location of chondrocytes and define the entire region of interest. Slides were first deparaffinized and hydrated in the same manner mentioned. Slides were then incubated at room temperature for 15 minutes in diluted proteinase K solution and then rinsed with phosphate-buffered saline (PBS). This was followed by 5 minutes of incubation in endogenous oxidation-blocking solution, 3% hydrogen peroxide, at room temperature and then rinsed in PBS. After that, slides were incubated at room temperature for 30 seconds in equilibration buffer, then in working strength TdT enzyme for an hour at 37°C. Slides were then incubated in working strength stop/wash buffer for 10 minutes at room temperature, and they were then rinsed in PBS and incubated in anti-digoxigenin conjugate for 30 minutes at 37°C. Slides were rinsed again in PBS and mounted with DAPI to stain the nuclei. The mean number of immunopositive cells was calculated for each group examining six to eight animals per group. The number of immunopositive cells was divided by the area or as a percentage of the total number of cells in the region of interest.

### 2.5 Cell culture, RNA extraction, qPCR

ATDC5 chondrocytes obtained from the American Type Culture Collection (ATCC) (Manassas, VA, USA) were used for *in vitro* experiments. Cells were cultured in 50% Dulbecco’s modified Eagle’s medium (DMEM) (Gibco, Gaithersburg, MD, USA) and 50% F12 (Gibco) with 5% fetal bovine serum (FBS) and 1% Antibiotic-Antimycotic (Anti-Anti) (Thermo Fisher Scientific, Waltham, MA, US). Hypertrophic differentiation induction was performed using ascorbic acid (50mg/mL) for 6 days with increasing concentrations of NaH2PO4 0.5mM, 1mM, and 2mM (37). All cell cultures were maintained in a 5% CO2 humidified incubator at 37°C.

Quick-RNA MicroPrep kit (Zymo Research, Irvine, CA, USA) was used according to the manufacturer’s instructions to isolate RNA. RNA was converted to cDNA using an ABI High- Capacity RNA-to-cDNA kit (Applied Biosystems; cat# 4387406). caspase3 mRNA levels were measured by qPCR in ATDC5 chondrocytes cultured in low glucose (5mM d-glucose) and high glucose (25mM d-glucose) for 5 days and transfected with FOXO1 siRNA or scrambled siRNA. qPCR was performed using ABI Fast SYBR Green Master Mix (cat# 4385612) and a StepOne Plus real-time PCR system (Applied Biosystems). Relative amounts were calculated using the ΔΔCt method. Data are expressed as percent input after quantitative amplification of equivalent amounts of DNA. Experiments were performed with triplicate replicates and carried out three times with similar results.

### 2.6 Dual-luciferase reporter

siRNA transfections with ATDC5s were performed at approximately 60-70% confluence in 6-well plates with 10nM siFOXO1 or scramble control (Dharmacon, Lafayette, CO) using GenMute transfection reagent (Rockville, MD) according to the manufacturer’s instructions. Plasmid transfections were performed in OptiMEM (Gibco) medium with 1250ng plasmid in 3.75ul of Lipofectamine 3000 transfection reagent per well and following manufacturer’s instructions with ATDC5s at approximately 60-70% confluence for 4.5 hours before being replaced with full media. To quantify caspase-3 expression, cells were co-transfected with treatment vectors: empty, ADA [Threonine 24 to Alanine (A) and Serine 253 to Aspartate (D) and Serine 316 to Alanine (A)], ADA + 6KQ (K242, K245, K259, K262, K271, and K291 were replaced with glutamine on ADA), ADA + 6KR (K242, K245, K259, K262, K271, and K291 were replaced with arginine on ADA), KQ(K242, K245, K259, K262, K271, and K291 were replaced with glutamine) or KR(K242, K245, K259, K262, K271, and K291 were replaced with arginine) mutants (Addgene, Cambridge, MA. pCMV5 backbone) along with a caspase-3 luciferase reporter (pGL4) as described and generously provided by Dr. Estrov (38). Co-transfection utilized the same transfection protocol with an expression vector, and the reporter was added at a 1:1 ratio of 150ng per well in a 48-well plate. Expression values were normalized using a Renilla control (pGL4) containing the CMV promoter at a 1:20 ratio. Results were quantified using a Dual-Luciferase Reporter Assay Kit from Promega (Madison, WI, cat# - E1960) and quantified on a Tecan Infinite M200. Experiments were performed with triplicate replicates and carried out three times with similar results.

### 2.7 Chromatin immunoprecipitation

Chromatin immunoprecipitation (ChIP) assays were performed with the ChIP-IT Kit (Active Motif, Carlsbad, CA) using approximately 1.5 x107 ATDC5 cells. The cells were cultured using multiple conditions, including 1) hypertrophic differentiation for 6 days using differentiation media as mentioned earlier; 2) cells at 70–80% treated with CML-BSA (200 mg/mL), an AGE, for 3 days or unmodified BSA (200 mg/mL) for a similar period; and 3) cells grown in High glucose (HG) (25 mmol/L) media for 5 days. Formaldehyde was used to fix the cells and nuclei obtained following Dounce homogenization. ChIP was performed following the manufacturer’s instructions using an anti-FOXO1 antibody (5 mg) (SC-11350X; Santa Cruz Biotechnology) or control polyclonal non-specific IgG (Cell Signaling Technology). Protein G-coupled beads were used to purify the chromatin–antibody complexes. Three quantitative real-time PCR reactions for the caspase-3 promoter region, which contains FOXO1 consensus response elements, were done with similar results. Experiments were performed with triplicate replicates and carried out three times with similar results.

## 3 Statistics

All data were analyzed by one-way analysis of variance and differences between groups determined using Tukey’s *post-hoc* tests unless otherwise stated. Student’s t-test was used in some *in vitro* experiments where only two groups were compared. P < 0.05 was considered statistically significant. Data are expressed as the mean ± SEM.

## 4 Results

### 4.1 Diabetes and vascularization

We have previously examined the effect of diabetes on angiogenesis during fracture healing by identifying blood vessels with an antibody to Factor VIII or CD31 (39). As shown in **Table 1**, which was adapted from (39), there was a 65%–80% increase in the number of blood vessels between day 10 and day 16 in normoglycemic mice (p < 0.05) (**Table 1**). Diabetes reduced the number of Factor VIII^+^ small and moderate-sized blood vessels by almost half (p >0.05) (**Table 1**). Mechanistically, this was related to the level of inflammation as shown by the rescue of diabetes-reduced neovascularization when a TNFα-specific inhibitor, pegsunercept (PEG), was applied.

**Table 1:** Diabetic-induced reduced angiogenesis is restored to normal levels upon insulin treatment and TNF α inhibition in diabetic fracture healing.

A			
Factor V111 + small Vessels per mm2			
Day 10	Normoglycemic	Diabetic	Diabetic + insulin
	518	265*	447^+^
Day 16	896	497*	764^+^
Factor V111 + Moderate Vessels per mm2			
Day 10	Normoglycemic	Diabetic	Diabetic + insulin
	214	110*	199^+^
Day 16	364	179*	322^+^

B			
CD31 + small Vessels per mm2			
Day 16	Normoglycemic	Diabetic	Diabetic + PEG
	107	58*	94^+^
CD31 + Moderate Vessels per mm2			
Day 16	Normoglycemic	Diabetic	Diabetic + PEG
	47	29*	53^+^

(1A) Factor VIII positive blood vessels were evaluated at Day 10 and 16 post-fracture by IHC in areas of new bone formation in normoglycemic, diabetic, and insulin-treated diabetic mice *Indicates a significant difference between normal and diabetic (P<0.05). + Indicates a significant difference between insulin-treated and untreated diabetic animals (P<0.05). (1B) CD31 immunopositive blood vessels were counted 16 days post fractures in areas of bone formation in normoglycemic, diabetic, and TNFα-specific inhibitor pegsunercept treated diabetic mice. *Indicates a significant difference between normal and diabetic (P<0.05). +Indicates a significant difference between PEG-treated and untreated diabetic animals (P<0.05). Original data found in ref (39).

**Figure 1:**
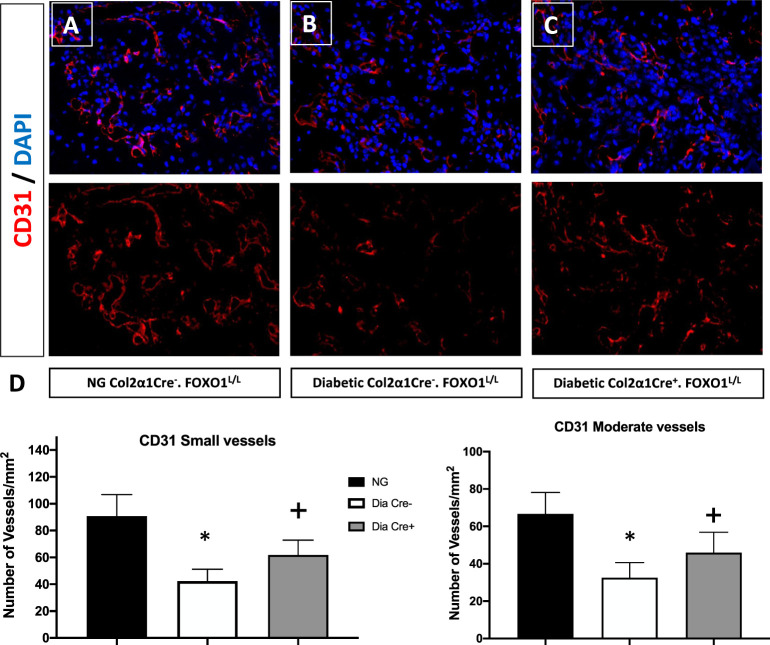
FOXO1 deletion in chondrocytes partially rescues diabetes impaired angiogenesis. Photmicrographs of histologic sections 16 days post fracture from **(A)** normoglycemic; **(B)** diabetic WT; and **(C)** diabetic FOXO1-deleted mice (Cre+.FOXO1LL). Immunofluorescence was carried out with CD31 specific-antibody to detect endothelial cells. No immunofluorescence was detected with control primary antibody (not shown). **(D)** Quantitative analysis of CD31 positive blood vessels in the transition zone. Blood vessels containing 2-4 CD31 positive cells were considered as small vessels while moderate-sized vessels contained 5-8 CD31 positive cells. Data are expressed as mean ± SEM. *indicates a significant difference between specimens from study group and matched normoglycemic animals. + indicates signficant difference between diabetic Cre-.FOXO1LL and diabetic Cre+.FOXO1LL groups. Significance was determined by ANOVA followed by Tukey’s *post-hoc* test (P<0.05).

Since we had shown that some of the effects of TNF could be directly related to the transcription factor FOXO1 (40), we examined the hypothesis that FOXO1 is a key factor in regulating angiogenesis in diabetic animals. This was accomplished by studying neovascularization in healing fractures in animals with chondrocyte-specific FOXO1 deletion in diabetic experimental (Col2a1Cre^+^.FOXO1^L/L^) compared to diabetic control (Col2a1Cre^–^.FOXO1^L/L^) littermates. Diabetes resulted in a ~ 50% reduction in the number of both small and moderately sized vessels compared to the WT (**Figure 1**, P > 0.05). Conditional FOXO1 deletion in chondrocytes in diabetic animals resulted in a partial and significant rescue of diabetes-inhibited formation of small and moderate-sized blood vessels (P<0.05).

### 4.2 Diabetes increases chondrocytes apoptosis via FOXO1-dependent mechanism

We have previously examined apoptotic cells in fracture healing using a TUNEL assay (40). **Table 2**, adapted from (40), demonstrated that the number of apoptotic chondrocytes was 5.4-fold higher in the diabetic group compared to the normoglycemic (P<0.05; **Table 2**). Insulin treatment significantly reduced most of this increase (P<0.05). When the entire callus was examined, the diabetic group showed a 2.5-fold increase in the total number of apoptotic cells compared to the normoglycemic, which was largely rescued by insulin treatment (P<0.05; **Table 2**). The increase in apoptosis was directly linked to the effect of TNF-α as demonstrated by a complete rescue when TNF-α was inhibited by pegsunercept. There was also no significant difference in the number of apoptotic chondrocytes between the diabetic and the normoglycemic group on day 10. Chondrocyte apoptosis increased further on day 16 post-fracture in the diabetic animals, and the increase was rescued to normal levels upon PEG treatment (P<0.05; **Table 2**.)

**Table 2:** Diabetes upregulated the number of apoptotic chondrocytes.

A			
Apoptotic chondrocytes/mm2 of cartilage	Normoglycemic	Diabetic	Diabetic + insulin
	0.5	2.7*	1^+^
Total Apoptotic cells/mm2 of callus	Normoglycemic	Diabetic	Diabetic + insulin
	1.8	4.2*	2^+^

B			
Day 10 Apoptotic chondrocytes/mm2 of cartilage	Normoglycemic	Diabetic	
	0.89	0.93*	
Day 16 Apoptotic chondrocytes/mm2 of cartilage	Normoglycemic	Diabetic	Diabetic + PEG
	1.17	2.43*	0.74^+^

(2A) Quantitative analysis of apoptotic cell numbers in normoglycemic, diabetic, and insulin-treated diabetic mice measured in TUNEL stained sections combined with safranin-O/fast green stain to distinguish chondrocytes. (2B) Sections from fracture calluses were examined by the TUNEL assay and counterstained with safranin-O/fast green to identify apoptotic chondrocytes in the cartilage area. Quantitative analysis of TUNEL-positive cells in diabetic mice, matched normoglycemic control mice, and diabetic mice were treated with pegsunercept (PEG) which was started 10 days after fracture. *Indicates a significant difference between normal and diabetic (P<0.05). + Indicates a significant difference between insulin-treated or pegsunercept-treated and untreated diabetic animals (P<0.05). Original data is found in ref (14).

Insulin treatment and TNFα inhibition rescued it to normal levels.

**Figure 2:**
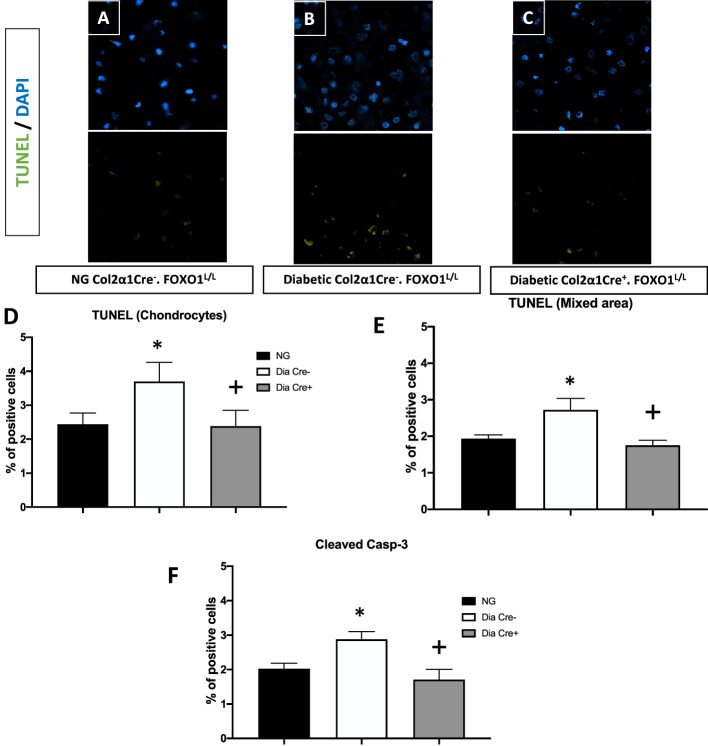
Diabetes significantly increases chondrocytes apoptosis, which is rescued by FOXO1 deletion in diabetic mice. Fracture sites were examined by a fluorescent TUNEL assay 16 days post fracture from **(A)** normoglycemic; **(B)** diabetic WT; and **(C)** diabetic FOXO1-deleted mice (Cre+.FOXO1LL). **(D, E)** Quantitative analysis of TUNEL positive cells in the cartilage area and transitional area containing both cartilage and bone, respectively. **(F)** Quantitative analysis of caspase-3 positive chondrocytes. Data are expressed as mean ± SEM. *indicates a significant difference between specimens from diabetic and matched normoglycemic animals; + indicates a significant difference between specimens from diabetic animals with FOXO1 deletion compared to littermate diabetic control animals. Significance was determined by ANOVA followed by Tukey’s *post-hoc* test (P<0.05).

We then examined whether the transcription of FOXO1 mediated apoptosis in diabetic fracture healing using the experimental animals described above. There was a ~ 50% increase in TUNEL-positive chondrocytes in the hypertrophic and the mixed zone that contains both cartilage and bone (**Figures 2A–E**, p<0.05). This increase was completely rescued to normal levels upon specific FOXO1 deletion in chondrocytes in diabetic animals (**Figure 2F**, p<0.05).

### 4.3 FOXO1 regulates caspase-3 under diabetic conditions

To further examine how diabetes could increase chondrocyte apoptosis, we measured the levels of cleaved caspase-3. *In vivo* immunofluorescence with an antibody specific for cleaved caspase-3 showed that diabetes increased caspase-3 positive chondrocytes by ~50% compared to fracture healing in normoglycemic animals (**Figure 2D**, p<0.05). FOXO1 deletion in chondrocytes in diabetic animals reduced the over-expression of caspase-3 to levels similar to that of the normal group (**Figure 2D**, P>0.05).

**Figure 3:**
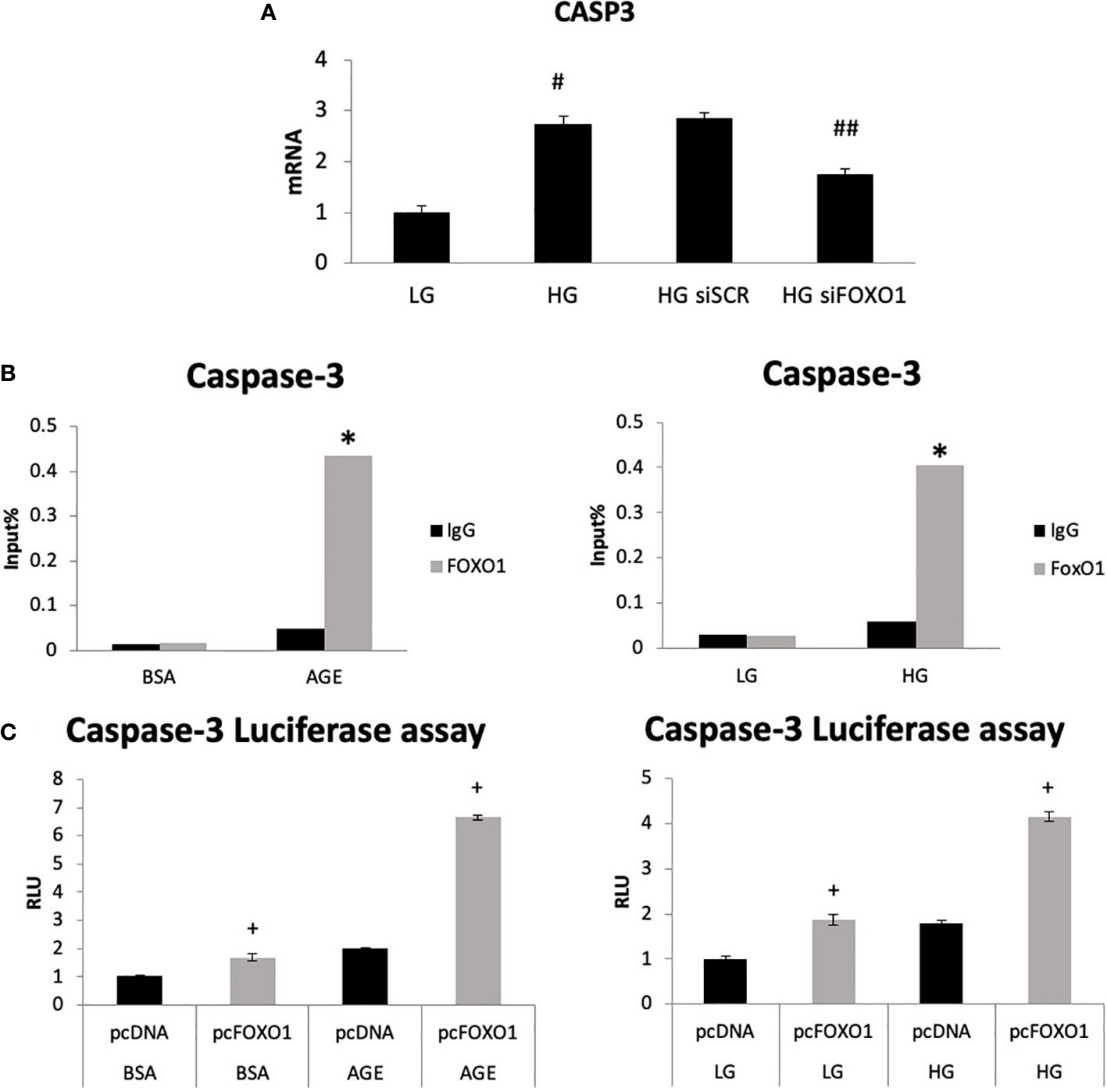
High glucose and an AGE modulate FOXO1 regulation of caspase-3. **(A)** Chondrocytes (ATDC5) were incubated in standard media (LG) or media supplemented with high glucose (HG) (25mM) for five days or 200ug/ml carboxymethylysine-modified BSA, an AGE or unmodified BSA for three days. Chromatin immunoprecipitation (ChIP) assays were performed by pull down with FOXO1 antibody or matched IgG. **(B)** Chondrocytes were co-transfected with a FOXO1 expressing plasmid and a caspase-3 luciferase reporter construct and incubated in standard media, HG media, and an AGE containing media. Luciferase activity was measured. **(C)** Chondrocytes were incubated in HG and transfected with siFOXO1 and or scrambled siRNA (siSCR). Caspase-3 mRNA level were analyzed by qPCR. Results are expressed as the mean ∓SE. *indicates p <0.05 compared to control IgG group. ^+^indicates p <0.05 compared to matched FOXO1 non-mutant. ^#^Indicates p <0.05 compared to LG group. ^##^indicates p <0.05 compared to siRNA control group.

We then directly assessed the role of FOXO1 in regulating caspase-3 expression by transfecting chondrocytes with FOXO1 siRNA or scrambled siRNA in low-glucose (LG) and high-glucose-containing media (HG). HG media resulted in a 2.7-fold increase in caspase-3 expression (**Figure 3A**, p<0.05). Silencing FOXO1 with siRNA significantly downregulated the increase in caspase-3 expression induced by HG. ChIP assays were undertaken to measure FOXO1 interaction with the caspase-3 promoter. HG stimulated an ~ 8-fold increase in FOXO1 binding to the caspase-3 promoter (**Figure 3B**, p<0.05). Treatment of chondrocytes with an advanced glycation end product (AGE), carboxymethyl lysine modified BSA, stimulated a similar increase. Luciferase reporter assays were undertaken to examine the direct effect of HG and AGE on caspase-3 promoter activity. Transfection of chondrocytes with a FOXO1 expression vector increased caspase-3 transcriptional activity 4-fold in the presence of HG compared to standard (LG) media and 6-fold in the presence of AGE compared to unmodified BSA (**Figure 3C**, p<0.05). Interestingly, the combination of HG or AGE with FOXO1 transfection was greater than the effect of FOXO1 transfection alone.

## 5 Discussion

Diabetic fracture healing is characterized by increased inflammation and oxidative stress, which reduces osteoblast proliferation and differentiation and interferes with the production of the osteoid matrix to limit the healing response (41). However, the role of chondrocytes in the healing has not been investigated as thoroughly. To better understand the mechanisms of how diabetes alters the healing, we examined the effect of diabetes on vascularization and apoptosis. We specifically looked at the regulatory role of the transcription factor FOXO1in chondrocytes in this process.

It is well known that diabetes impairs angiogenesis and wound healing (42). Our results indicate that diabetes significantly reduces the number of vessels in the fracture callus, as shown by the reduction in CD31-positive small and moderately-sized vessels studied in histologic samples (39). The impact of diabetes was directly related to the level of inflammation as inhibition of TNF rescued this effect. We add to this information by showing that FOXO1 also plays an important role in interfering with diabetic fracture healing. Reduced angiogenesis is clinically important since it can lead to delayed repair, as shown in an animal model with an ischemic fracture that impairs healing. (43). These results were unexpected since in normal healing, FOXO1 expression in chondrocytes leads to enhanced angiogenesis through the production of VEGFA (31). The downstream mechanisms that may account for the improvements in angiogenesis when FOXO1 is deleted in chondrocytes may be explained by a recent finding that FOXO1 deletion leads to increased inflammation in the fracture callus in diabetic animals (26). Thus, in normal fracture healing, FOXO1 may promote angiogenesis through the production of VEGFA, as shown in both soft tissue and hard tissue healing responses (26, 44). In contrast, FOXO1 in diabetic conditions has a shift in downstream targets manifested by a reduction in growth factors and an increase in inflammatory mediators that interfere with healing responses (45).

Diabetes also has a significant impact on apoptosis. A mechanism through which diabetes inhibits the early steps of fracture repair is due to the loss of mesenchymal stem cells to apoptosis caused by diabetes-enhanced inflammation (46). The increased apoptosis in bone linked to diabetes is significant since inhibiting apoptosis with a caspase-3 inhibitor or a TNF inhibitor results in significantly enhanced tissue formation in diabetic animals (9, 47, 48). FOXO1 deletion in chondrocytes reversed the effect of diabetes on apoptosis to normal levels. The apoptosis phase of the hypertrophic chondrocytes is a critical phase during fracture repair (2, 40, 49). There is a direct relationship between the vasculature, rate of apoptosis, and resorption of cartilage. It has been proposed that this process is mediated in part by the tumor necrosis factor alpha (TNF-α). TNF-α enhances the apoptosis of chondrocytes and upregulates the levels of pro-resorptive cytokines that regulate the remodeling phase by osteoclasts (50). Since FOXO1 is downstream of TNF-α, it is likely that activation of FOXO1 in diabetic fracture healing through FOXO1 activation leads to a dysregulation that impairs the healing process. Diabetes significantly upregulated more than 13 apoptotic pathways, including the caspase pathway (**Table 3**). Our findings here support the concept that FOXO1 upregulates caspase-3 activity in diabetic fractures and that it is transcriptionally regulated by increased FOXO1 binding to the caspase-3 promoter when chondrocytes are exposed to high levels of advanced glycation end products or high glucose levels. Additionally, the transcriptional activity of caspase-3 was directly enhanced by transfection with a FOXO1 expression vector that was significantly enhanced when chondrocytes were incubated in media supplemented with high glucose or AGEs. Apoptosis of chondrocytes may not only limit the formation of the cartilage matrix but lead to greater matrix degradation (51). The increased apoptotic activities may also lead to premature cartilage removal, which has been shown to impair the healing process. And lastly, chondrocyte transdifferentiation into osteoblasts may be inhibited by a loss of chondrocytes through apoptosis, which could ultimately reduce the formation of a bony callus during the endochondral process (52, 53). Based on those observations, the upregulated apoptotic activities can indirectly retard the healing by a number of different mechanisms.

**Table 3:** Apoptosis-related pathways upregulated in diabetic fracture healing.

Apoptotic gene sets	Upregulated by Diabetes
Passerini apoptosis	Yes
Apoptosis Kegg	Yes
Apoptosis	Yes
Death pathway	Yes
Vanasse BCL2 targets	Yes
NF-kB pathway	Yes
TNF and FAS network	Yes
TNFA NF-kB dep up	Yes
ST FAS signaling pathway	Yes
Caspase pathway	Yes
PKC pathway	Yes
Passerini oxidation	Yes
JNK up	Yes

Gene set enrichment analysis (GSEA) identified apoptosis-related pathways that were significantly upregulated. Original data is found in ref (40).

To answer whether FOXO1 activities in chondrocytes represent crucial mechanisms for impaired diabetic long-bone healing, we tested mice with lineage-specific FOXO1 deletion. FOXO1 deletion rescues reduced callus formation caused by diabetes measured by microCT and histologically (3). The mechanical properties of the calluses followed the same pattern. The maximum torque was reduced due to diabetes by ~ 70%, stiffness by 56%, toughness by 74%, and shear modulus by 60%. FOXO1 deletion restored these parameters of mechanical strength to normal levels in diabetic fractures.

In summary, we report here for the first time the important role of FOXO1 and chondrocytes in diabetic fracture healing by inhibiting angiogenesis during the fracture healing process. This contrasts with the positive role that FOXO1 has in promoting angiogenesis in normal animals (31). In addition, we show that FOXO1 in diabetic fracture healing also has a negative effect on increasing chondrocyte apoptosis.

## Data availability statement

The original contributions presented in the study are included in the article/supplementary files, further inquiries can be directed to the corresponding author/s.

## Ethics statement

The animal study was reviewed and approved by IACUC Committee of the University of Pennsylvania.

## Author contributions

MA: Experimental design, carrying out experiments, data interpretation, writing and revising the manuscript, and preparation and revision of figures. DG: Overall project and experimental design, data interpretation, writing and revising the manuscript, revision of figures, grant funding to support experiments. All authors contributed to the article and approved the submitted version.
